# Impaired iloprost-induced platelet inhibition and phosphoproteome changes in patients with confirmed pseudohypoparathyroidism type Ia, linked to genetic mutations in *GNAS*

**DOI:** 10.1038/s41598-020-68379-3

**Published:** 2020-07-09

**Authors:** Frauke Swieringa, Fiorella A. Solari, Oliver Pagel, Florian Beck, Jingnan Huang, Marion A. H. Feijge, Kerstin Jurk, Irene M. L. W. Körver-Keularts, Nadine J. A. Mattheij, Jörg Faber, Joachim Pohlenz, Alexandra Russo, Connie T. R. M. Stumpel, Dirk E. Schrander, Barbara Zieger, Paola E. J. van der Meijden, René P. Zahedi, Albert Sickmann, Johan W. M. Heemskerk

**Affiliations:** 1 Department of Protein Dynamics, Leibniz Institute for Analytical Sciences-ISAS-E.V., Dortmund, Germany; 2 Department of Biochemistry, CARIM, Maastricht University, PO Box 616, 6200 MD Maastricht, The Netherlands; 3 Sections of Pediatric Hematology and Endocrinology, Haemostaseology, Children’s Hospital, University Medical Center of Johannes Gutenberg University Mainz, Mainz, Germany; 4 Center for Thrombosis and Haemostasis (CTH), University of Mainz, Mainz, Germany; 5 Department of Clinical Genetics, Maastricht University Medical Centre, Maastricht, The Netherlands; 6 GROW-School for Oncology and Developmental Biology, Maastricht University Medical Centre, Maastricht, The Netherlands; 7 Department of Orthopedic Surgery and CAPHRI (Care and Public Health Research Institute), Maastricht University Medical Centre, Maastricht, The Netherlands; 8 Division of Pediatric Hematology and Oncology, Department of Pediatrics and Adolescent Medicine, Faculty of Medicine, Medical Center-University of Freiburg, University of Freiburg, Freiburg, Germany

**Keywords:** Molecular biology, Molecular medicine

## Abstract

Patients diagnosed with pseudohypoparathyroidism type Ia (PHP Ia) suffer from hormonal resistance and abnormal postural features, in a condition classified as Albright hereditary osteodystrophy (AHO) syndrome. This syndrome is linked to a maternally inherited mutation in the *GNAS* complex locus, encoding for the GTPase subunit Gsα. Here, we investigated how platelet phenotype and omics analysis can assist in the often difficult diagnosis. By coupling to the IP receptor, Gsα induces platelet inhibition via adenylyl cyclase and cAMP-dependent protein kinase A (PKA). In platelets from seven patients with suspected AHO, one of the largest cohorts examined, we studied the PKA-induced phenotypic changes. Five patients with a confirmed *GNAS* mutation, displayed impairments in Gsα-dependent VASP phosphorylation, aggregation, and microfluidic thrombus formation. Analysis of the platelet phosphoproteome revealed 2,516 phosphorylation sites, of which 453 were regulated by Gsα-PKA. Common changes in the patients were: (1) a joint panel of upregulated and downregulated phosphopeptides; (2) overall PKA dependency of the upregulated phosphopeptides; (3) links to key platelet function pathways. In one patient with *GNAS* mutation, diagnosed as non-AHO, the changes in platelet phosphoproteome were reversed. This combined approach thus revealed multiple phenotypic and molecular biomarkers to assist in the diagnosis of suspected PHP Ia.

## Introduction

Pseudohypoparathyroidism (PHP) characterises a heterogeneous group of disorders, of which the common feature is an end-organ resistance to parathyroid hormone. Patients diagnosed with pseudohypoparathyroidism type Ia (PHP Ia, OMIM: #103580) or with the related disorder PHP Ic also exhibit resistance to other hormones and show a variable set of clinical features, including as short stature, obesity, round face, subcutaneous ossification, brachydactyly, and other skeletal anomalies. Some patients also have mental retardation. Jointly these characteristics are classified as Albright hereditary osteodystrophy (AHO) syndrome. This infrequent and serious syndrome occurs with an estimated prevalence of 1:250,000, but it is likely underdiagnosed because of variation in the presentation and severity of the disease. In the past, PHP was also deduced from a defective activity of the erythrocyte Gsα protein, but the suitability of this test has been questioned^1^.

Consistent among patients with established PHP (including AHO) is impairment in the Gsα-mediated signalling effects of tissues in response to multiple hormones^2^. This impairment is linked to dysfunctional mutations in the *GNAS* complex locus, encoding for the Gsα protein. The imprinted gene complex locus *GNAS* is located on chromosome 20q13.2-13.3^3,4^. Importantly, genetic variation in this locus can also link to an altered regulation of blood pressure and an increased risk of cardiovascular disease^5^. In case of AHO, the syndrome characteristics are inherited from the mother, implicating that the affected tissues may preferentially express the maternal *GNAS* allele^6^. However, the disease can also be due to epigenetic variation^6^, in which case *GNAS* methylation studies are needed for a proper diagnosis of PHP patients^7^. On the other hand, paternal transmission of the mutated *GNAS* allele is not accompanied by hormone resistance, and is then classified as pseudopseudohypoparathyroidism (PPHP).

In platelets like in other cells, the stimulation of G protein-coupled receptors (GPCR), interacting with the Gsα β/γ complex, causes activation of adenylyl cyclase (AC), which enzyme produces the second messenger cAMP^8^. Elevation in cAMP triggers intracellular signalling events via the broad-spectrum, cAMP-dependent protein kinase A (PKA)^9^. The platelet Gsα-AC-PKA pathway is by far most strongly triggered via the IP receptor (*PTGIR*)^10^, which causes global inhibition of platelet adhesion, shape change, cytoskeletal changes, secretion, aggregation and procoagulant activity^11,12^. The endothelium-derived prostaglandin I_2_ (prostacyclin, stable mimetic iloprost) provides the main high-affinity trigger of IP receptors, along with the stable prostanoid prostaglandin E_1_ (PGE_1_) showing a somewhat lower affinity^13^. The other high-affinity receptor for prostacyclin, EP1, is not expressed on platelets^14^. Accordingly, both iloprost and PGE_1_ elevate platelet cAMP levels via Gsα, activate PKA and establish suppression of key signalling events including Ca^2+^ fluxes and integrin α_IIb_β_3_ activation^15,16^. Earlier studies describe that in patients with PHP Ia (AHO) the Gsα functional defect is indeed linked to lower platelet responses to both iloprost and PGE_1_^17^.

In our previous work, we have used protein mass spectrometry techniques for quantitative analysis of the global platelet proteome^18^ and of the iloprost-induced platelet phosphoproteome^19^. In the platelets from healthy individuals, the (phospho)proteome appeared to be markedly stable. This offers the possibility to exploit the platelet proteome to find abnormalities on the protein level in patients with a congenital deficiency or other pathologies^20,21^.

Here, we studied seven rare patients of four families with confirmed or suspected PHP Ia, which is the largest cohort examined so far for this rare syndrome. In the platelets from these patients, we determined altered responses of the Gsα-AC-PKA pathway using various multiparameter function tests, and compared these with quantitative changes in the PKA-dependent (phospho)proteome. These proteome changes were: (1) linked to a defective Gsα and PKA activity, (2) related to changes in platelet function, and (3) evaluated for the potential of discriminating between AHO and non-AHO.

## Results

### Impairment of Gsα-dependent responses in platelets from patients with suspected PHP Ia

**I**n order to assess abnormalities of the Gsα-AC-PKA pathway, we analysed the platelets from seven rare patients (four families) with confirmed or suspected PHP Ia (Table 1). Family I patient (mother P1) had a heterozygous, deleterious mutation in exon 1 of *GNAS*. Family II (mother P2 and two children P3–4) had a heterozygous, dysfunctional mutation of *GNAS* in exon 3. Family III with unaffected mother P5 included a child (P6), showing all physical characteristics of PHP Ia, while no genetic mutation was found so far. Family IV consisted of a child (P7) with a heterozygous deleterious mutation in the *GNAS* locus and a differential diagnosis including PHP. On the days of measurement, blood samples were also obtained from healthy control subjects (C1–12). Throughout this paper, the numbering of individual patients and controls has been kept the same.

**Table 1:** Investigated families and patients with symptomatic or suspected PHP Ia.

	Patient	Diagnosis	Mutation in *GNAS* locus	Platelet count L^-1^
Family I	P1	PHP type Ia	c.1A > G (p.Met1Val)	226 × 10^9^
Family II	P2 (mother P3,4)	PHP type Ia	c.338G > C (p.Lys338Asn)	215 × 10^9^
	P3	PHP type Ia	c.338G > C (p.Lys338Asn)	173 × 10^9^
	P4	PHP type Ia	c.338G > C (p.Lys338Asn)	244 × 10^9^
Family III	P5 (mother P6)	(asymptomatic)	not found	255 × 10^9^
	P6	PHP type Ia	not found	214 × 10^9^
Family IV^a^	P7	PHP suspected	c.565_568del	278 × 10^9^

Listed are patients with (suspected) PHP Ia (OMIM: 103,580), confirmed mutations and whole blood platelet counts. Limited blood was obtained from P7 (young child).

^a^Parents did not allow further examination.

**Figure 1:**
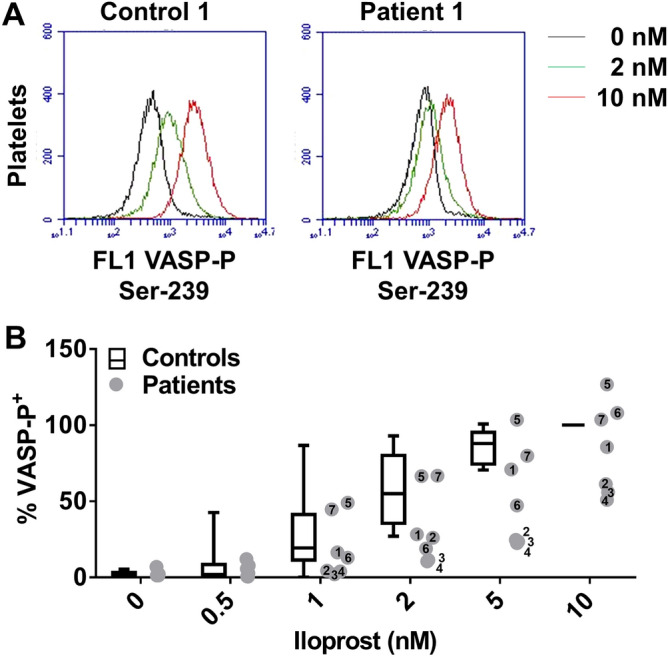
*Changes* *in* *Gsα-mediated* *VASP* *phosphorylation* *in* *platelets* *from* *patients* *with* *suspected* *PHP* *Ia*. Isolated, washed platelets were preincubated with iloprost (0–10 nM), fixed, permeabilised and stained with FITC anti-P-VASP mAb. (**A**) Representative flow cytometric histograms of VASP phosphorylation after iloprost treatment of platelets from control subject C1 and patient P1. (**B**) Quantified VASP phosphorylation results from control subjects (C1–12) and patients (P1–7). Box plots indicate medians ± interquartile ranges (whiskers represent 2.5–97.5th percentiles, *n* = 12).

Isolated platelets from all seven patients (P1–7) and 12 control subjects (C1–12) were used for the assessment of iloprost-induced VASP phosphorylation at Ser-239, which is a golden standard method to establish affected PKA-dependent phosphorylation events^22^. For this purpose, platelets were treated with a range of iloprost concentrations (0.5–10 nM) for 1 min, i.e. a time point reflecting the early phosphorylation activity of PKA. In the cells from control subjects, VASP phosphorylation increased dose-dependently with limited inter-individual variation (Fig. 1A,B). In the platelets from patients P1-4,6 (families I–III), VASP phosphorylation was impaired at a variable degree, in that higher doses of iloprost were needed to reach the phosphorylation level seen in platelets from the control group. Exceptions were the platelets from the unaffected patient P5 and the atypical patient P7, which showed a normal dose response of iloprost-induced VASP phosphorylation.

In the diagnostics laboratory, light transmission aggregometry (LTA) is commonly used to monitor platelet function abnormalities, requiring the use of day control samples^23^. The LTA method can reveal defects in Gsα signalling activity, by establishing the inhibitory effects of IP receptor agonists iloprost or PGE_1_ on collagen-induced platelet aggregation^17^. From four patients, sufficient blood could be obtained to measure platelet aggregation responses. In the platelets from the day-control subjects, collagen-induced aggregation was fully inhibited by 100 nM PGE_1_ (Suppl. Fig. 1A). In the platelets from patient P1 (family I), PGE_1_ was less inhibitory; in that 300 nM still caused appreciable aggregate formation. A similar defective response to PGE_1_ was seen in response to platelet aggregation with stable ADP, PAR1 agonist or convulxin (Suppl. Fig. 1B). For the other patients, the platelets from P2 (family I) and P5 (family III) showed a similar impairment, again requiring an increased dose of PGE_1_ to abrogate collagen-induced aggregation (Suppl. Fig. 1C). In contrast, the platelets from P5 responded similarly as the control platelets. For patient P1, sufficient platelets were isolated to confirm low inhibition of aggregation also with iloprost (Suppl. Fig. 1D), and to establish an impairment in Gαs-dependent elevation in cytosolic cAMP (Suppl. Fig. 1E,F). Taken together, these data indicate that the platelets from patients P1–4 and P6 (but not the asymptomatic P5) show a variably impaired response to PGE_1_ and iloprost in terms of low cAMP-dependent VASP phosphorylation and aggregation inhibition.

### Impairment of Gsα-mediated thrombus formation in whole blood from patients with suspected PHP Ia

**Figure 2:**
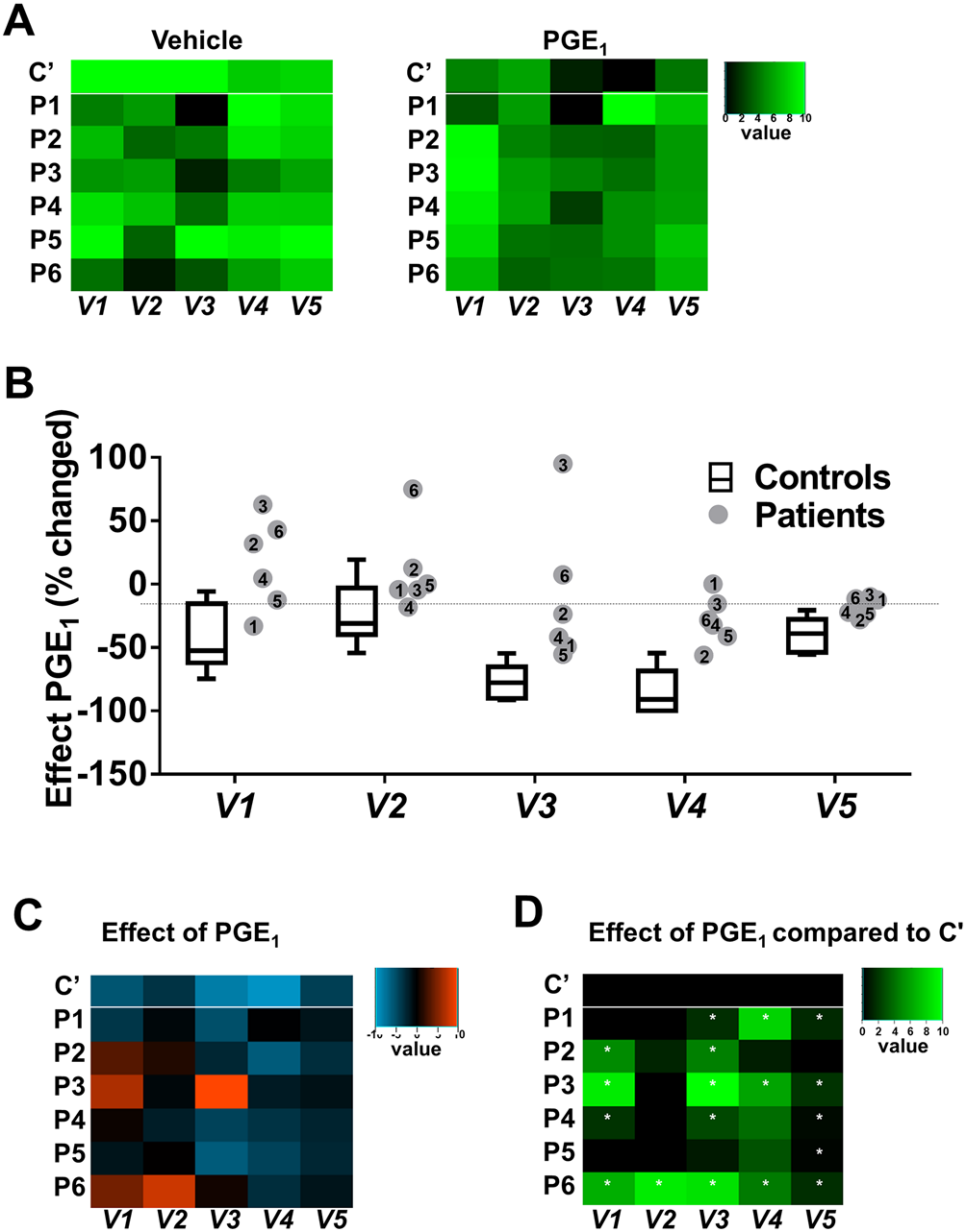
*Changes* *in Gsα-mediated inhibition of* *thrombus* *formation* *in* *blood* *from* *patients* *with* *suspected* *PHP* *Ia.* Whole blood from control subjects (C1–8) and patients (P1–6) was perfused over collagen at wall shear rate of 1,000 s^−1^ for 4 min. Thrombi formed were evaluated from brightfield microscopic images and fluorescence images (staining with FITC anti-P-selectin mAb). Blood samples were preincubated with vehicle or PGE_1_ (100 nM), as indicated. (**A**) Unit variance normalised parameters of thrombus formation (0–10): platelet adhesion (*V1*); P-selectin expression (*V2*); aggregate integrated feature size (*V3)*; thrombus multilayer score (*V4*); and thrombus morphological score (*V5*). Heatmaps show median values for all control subjects (C1–8, C’) and values for individual patients (P1–6) of blood flow runs in the presence of vehicle (left) or PGE_1_ (right). (**B**) Quantified effect of PGE_1_ on thrombus parameters; data for control subjects indicated as medians ± interquartile ranges (whiskers represent 2.5–97.5th percentiles, *n* = 8). (**C**) Heatmap of normalised effects of PGE_1_ per parameter and patient. (**D**) Subtraction heatmap of PGE_1_ effects in comparison to means of control platelets, *p < 0.05 (one-way ANOVA).

Microfluidic assessment of collagen-dependent thrombus formation in flowing whole blood provides an overall assessment of platelet functions^24^. To determine a role of Gsα in this assay, whole blood samples from patients and control subjects were preincubated with vehicle or PGE_1_, and then flowed at defined wall shear rate^25^. The thrombi formed on collagen were analysed for variables, indicative of the platelet activation state: platelet adhesion (*V1*), P-selectin expression (*V2*), platelet aggregation as integrated feature size (*V3*), a thrombus multilayer score (*V4*), and a thrombus morphological score (*V5*). Blood samples from control subjects indicated that 100 nM PGE_1_ was sufficient to cause an ~ 50% overall reduction in thrombus formation. Hence, this PGE_1_ concentration produced smaller-sized thrombi (*V3,4)*, which was accompanied by lower platelet adhesion (*V1*) and lower platelet secretion *(V2*) (Fig. 2). The other Gsα-stimulating agent iloprost induced similar effects as PGE_1_ (Suppl. Fig. 2).

Application of the flow assay with blood samples from patients P1-6 and consecutive control subjects (C1-8) resulted in a reducing effect of PGE_1_ for all variables, although we noted some differences between the patients. This was illustrated in a heatmap (Fig. 2A), constructed of the normalised variables (range 0–10) per patient, in comparison to the averaged data from control subjects, using an earlier used procedure^26^. This assessment of PGE_1_ effects on thrombus formation pointed to largest deviations for patients P2,3,6, when compared to normal ranges (C1–8) (Fig. 2B). Visualisation in a heatmap thus showed the effects of PGE_1_ per variable and patient, in comparison to the average control data after a applying a statistical filter of *p* < 0.05 (Fig. 2C,D). These flow data revealed that the inhibitory effect of PGE_1_ is reduced for most thrombus variables in patients P3,6, and for two/three variables in patients P1,2,4. In contrast, values for P5 were largely within the normal ranges. Taken together, these findings pointed to a consistent impairment in PGE_1_-mediated suppression of whole blood thrombus formation for patients P1-4,6, but not for the asymptomatic patient P5.

### Minor changes in global proteome of patient platelets

By combined analysis of the proteome and phosphoproteome^27^, we examined the protein composition of available platelets from all patients with suspected PHP Ia (P1–4,6,7), again in comparison to the day control subjects (C1–4). Directly after isolation, the purified platelets were incubated with 0, 2 or 10 nM iloprost for 1 min at 37 °C, i.e. a condition known to identify the iloprost/PKA phosphoproteome^19^.

**Figure 3:**
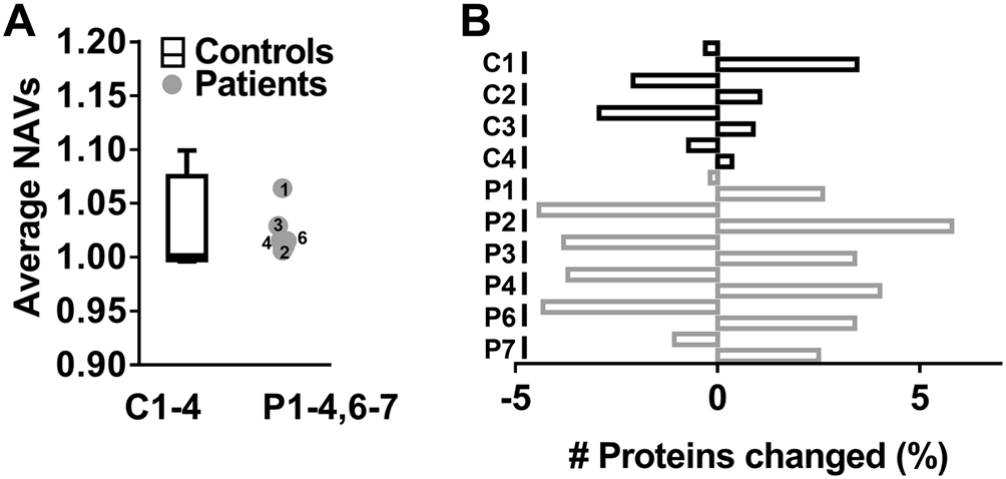
*Minor alterations in global platelet proteome in patients with suspected PHP Ia.* Global proteomics analysis of platelets from day control subjects (C1–4) and patients P1–4,6,7, providing quantitative information on 1,651–3,917 unique proteins. (**A**) Averaged normalised abundance ratios over all identified proteins per control subject, presented as medians ± interquartile ranges (whiskers indicate 2.5–97.5th percentiles). (**B**) Percentage factions of altered proteins per subject in comparison to mean of controls, C’); defined as values outside range of log2 − 0.322 to + 0.322.

Because of the use of iTRAQ or TMT labels, proteome analysis of the platelets was performed in four sets in 8-plex or 10-plex measurements, respectively (Suppl. Table 1). Mass spectrometry of the trypsin-treated platelet lysates provided quantitative information on 1,651 (C1, P1), 3,917 (C2, P2,6), 3,859 (C3, P3,4), and 1,957 (C4, P7) unique proteins (Suppl. Datafile S1). To assess for relevant differences in protein abundance between platelet preparations, we applied cut-off ratios outside the range of − 0.322 to 0.322 (log2 transformed), representing ≥ 25% up- or downregulation. Mean normalised abundance values (NAVs) for all proteins, analysed per set of samples, were grossly within normal ranges for all patients (Fig. 3A). Importantly, for the individual control subjects, small proportions of about 1–3% of the identified proteins showed ≥ 25% down- or upregulation (Fig. 3B). This small variation of the platelet proteome of healthy subjects is well in-line with our previous papers. For the individual patients P2,3,4,6 these percentages increased up to 4–5%. We hence concluded that the analysed global platelet proteomes of controls and patients were comparable.

### Assessment of iloprost-induced, Gsα-dependent phosphoproteome in control platelets

Phosphoproteomics analysis of the iTRAQ- or TMT-labelled lysates from resting and iloprost-treated (2 or 10 nM) platelets was performed after TiO_2_ enrichment. The total numbers of phosphopeptides quantified were 3,457 (C1, P1), 4,845 (C2, P2,6), 5,540 (C3, P3,4), and 3,812 (C4, P7) (Suppl. Datafile S1). This corresponded per sample set to 1,164, 1,599, 1,676 and 1,361 unique proteins, respectively.

To establish the relevant iloprost-induced changes, we first listed the phosphopeptides that were present in multiple, ≥ 3 control samples (C1–4). For the found 2,516 phosphopeptides, the same cut-off was used as above, in order to define relevant changes induced by 2 nM iloprost (in brackets 10 nM iloprost), i.e. with ≥ 25% up- or downregulation. This provided an overall list of 453 regulated phosphopeptides, of which 146 (263) were upregulated and 192 (190) were downregulated (Suppl. Datafile S2). This represents a 50% increase in comparison to the earlier reported 299 iloprost-regulated phosphopeptides^19^.

**Figure 4:**
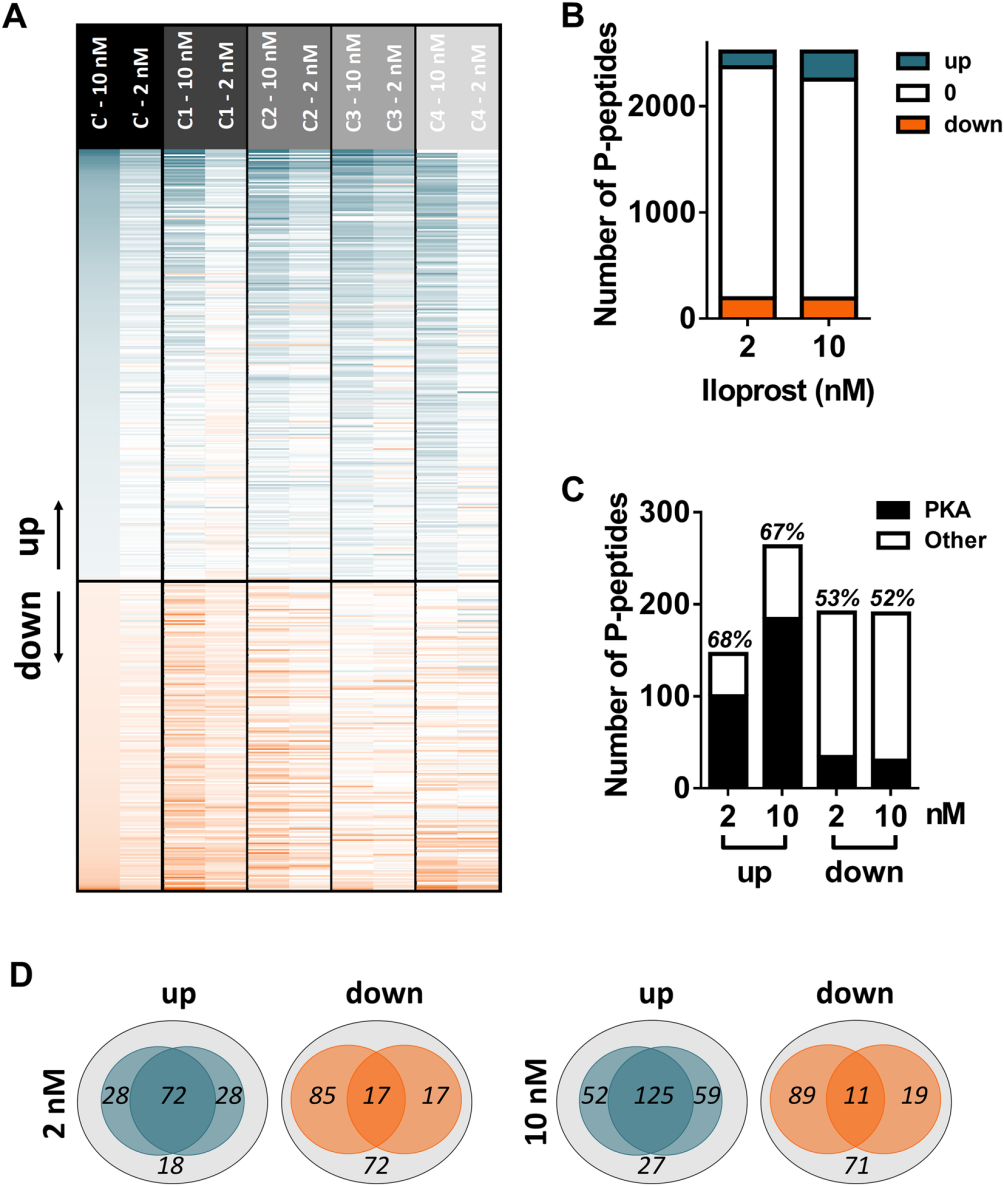
*Iloprost-induced and PKA-mediated changes in phosphoproteome of control platelets.* (**A**) Heatmap of 453 phosphopeptides assigned as upregulated (blue) or downregulated (orange) by 10 or 2 nM iloprost in control platelets (C1–4). Left lanes: mean effects in platelets from the control subjects (C’). Phosphopeptides were ordered according to mean effect size by 10 nM iloprost. Next lanes: iloprost effects for the control subjects. Relevant regulated changes were arbitrarily thresholded for outside range of log2 − 0.322 to + 0.322. (**B**) Numbers of phosphopeptides identified as up- or downregulated by iloprost in ≥ 3 control subjects. (**C**) Fractions of phosphopeptides identified as up- or downregulated with a PKA consensus site; percentages refer to overlap with previous identification^19^. (**D**) Venn diagrams presenting relevant up- and downregulated proteins by 2 or 10 nM iloprost. Left circles: previously identified, right circles: positive PKA consensus site.

By ordering the regulated phosphopeptides according to mean changes in (10 nM stimulated) control platelets, we importantly obtained highly similar patterns of iloprost-induced changes for the individual control subjects (Fig. 4A). Raising the iloprost dose from 2 to 10 nM resulted in more upregulated phosphopeptides, as expected (Fig. 4B). At either iloprost concentration, the majority of upregulated phosphopeptides (68–70%) were found to contain a consensus PKA phosphorylation site, whereas only few of the downregulated phosphopeptides (16–18%) contained such sites (Fig. 4C). The Venn diagrams of Fig. 4D illustrate this by showing a larger overlap of the upregulated than of downregulated phosphoproteins with PKA consensus site.

Assignment of the 453 phosphopeptides (regulated by 2–10 nM iloprost) to platelet function classes indicated a similar pattern for the up- and downregulated sites (Table 2). In general, the higher dose of 10 nM iloprost gave an expected higher number of upregulated (not downregulated) phosphopeptides. Those function classes that comprised (phospho)proteins that were most frequently modified included: signalling & adapter proteins (13.0–19.0%), cytoskeleton actin-myosin (9.5–13.7%), protein kinases & phosphatases (9.1–14.6%), and small GTPases and regulators (8.9–14.4%).

**Table 2:**
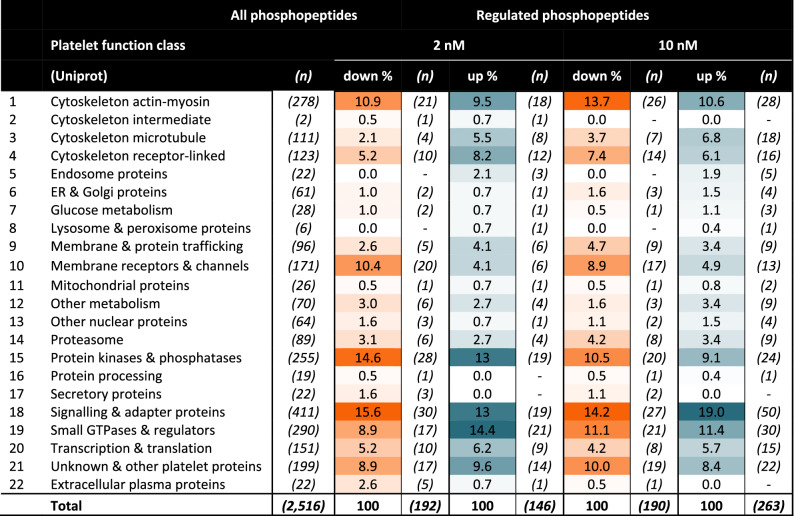
Iloprost-induced changes in phosphoproteome and platelet functions.

All 2,516 phosphopeptides of the iloprost phosphoproteome, identified in control platelets, were assigned to 22 platelet function classes, according to Uniprot. Indicated per class are the fractions of phosphopeptides identified as up- or downregulated by 2 or 10 nM iloprost, in platelets from ≥ 3 control subjects (C1–4). Note that cut-off levels were used per column to define downregulation or upregulation, as for Fig. 4. Indicated also are numbers (*n*) of altered phosphopeptides per function class.

To further assess the signalling mechanisms altered by iloprost, we performed a Reactome pathway analysis. Taking as input the iloprost-regulated proteins, this again showed a high similarity in pathways that were covered by upregulated or downregulated proteins. Reactome thus identified pathways of signal transduction, haemostasis, cell–cell interactions, and platelet activation (Suppl. Table 2). As an alternative approach, we furthermore performed pathway analysis using the Gene Ontology resource. Again, the most abundant specific pathways were those of: response to stimulus, signal transduction, cytoskeleton organization, regulation of phosphorylation and haemostasis (Suppl. Table 3). These pathway analyses thus support a coordinated mechanism of iloprost-induced platelet inhibition via Gsα-AC-PKA signalling to modulate a wide range of platelet haemostatic responses.

### Overall phosphoproteome changes by iloprost in patient platelets

**Figure 5:**
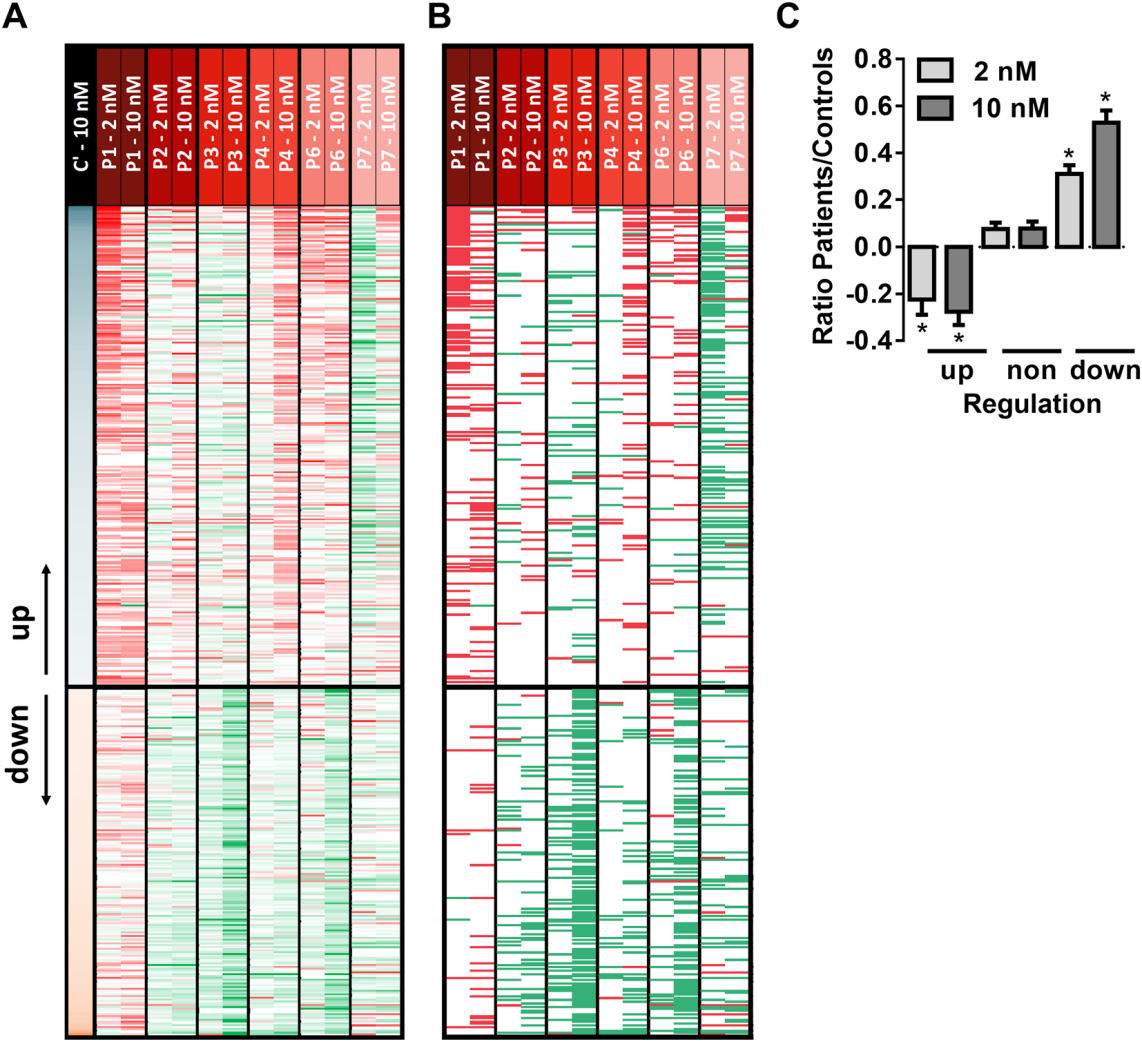
*Iloprost-induced changed in phosphoproteome of platelets from patients with suspected PHP Ia.* (**A**) Heatmap of 453 platelet phosphopeptides identified as increased (green) or decreased (red) at indicated iloprost concentration per patient in comparison to mean effect in 3 or 4 control subjects (C1–4, C’). Left lane: mean effect of 10 nM iloprost in control platelets (see Fig. 4A). (**B**) Heatmap as in panel A, but restricted to relevant differences, filtered for outside control range of log2 2× (− 0.322 to + 0.322). (**C**) Average ratios of iloprost effects in platelets from selected patients (P1–4,6) *versus* control subjects (C1–4) regarding top-100 up-, non-, and downregulated phosphopeptides.*p < 0.05 vs. controls (2-sided t test).

In the peptide panels of platelets from all patients, stimulated or not with iloprost, we then searched for consistent changes in phosphorylation patterns, in comparison to the means of control platelets. For the 453 regulated phosphopeptides, this resulted in a heatmap of ratio differences per patient (Fig. 5A). After filtering for relevant changes (outside normal range of 2x − 0.322 to + 0.322; log2 values), we identified a list of iloprost-modulated peptides, which were consistently reduced in the platelets from P1,2,4,6 (Fig. 5B). However, these peptides were less deviant in P3 and were changed in the opposite way in P7. Calculation of the mean differences in the top-100 up- and down-regulated phosphopeptides between controls and patients P1,2,4,6 showed a consistent decrease in iloprost-upregulated phosphopeptides and a converse increase in iloprost-downregulated phosphopeptides in the patients' platelets (Fig. 5C). Counting the numbers of phosphopeptides with relevant changes in comparison to control subjects, resulted in an order of P1,6 > P2,4 > P3 > P7 (Table 3). Typically, for patient P7, the numbers of phosphopeptides with *in*creased phosphorylation exceeded those with decreased phosphorylation.

**Table 3:**
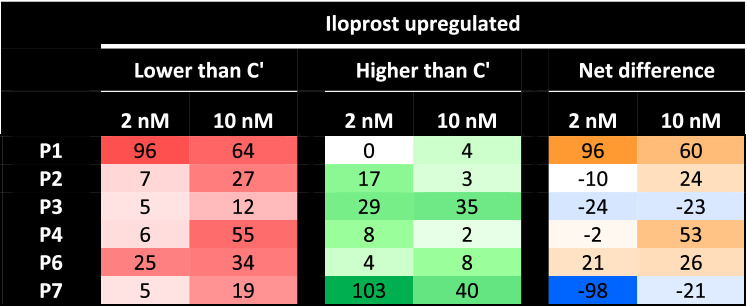
Relevant changes in iloprost upregulated phosphopeptides in platelets from individual patients.

Phosphopeptides classified as upregulated by 2 nM (146) or 10 nM (263) iloprost, and compared per patient (P1–4,6,7) versus mean values of controls (C’, C1–4). As relevant changes were considered all ratios outside the normal range of log2 2× (− 0.322 to + 0.322). Listed per patient are numbers of phosphopeptides lower or higher than normal ranges. Colour code of upregulated phosphopeptides: red, decreased compared to controls; white, unchanged; green, increased.

As further confirmation we evaluated how many of altered phosphopeptides contained a PKA consensus site. Histograms indicated a highly significant increase in mean NAVs after stimulation with 2 or 10 nM iloprost (*p* < 0.0001) for those phosphopeptides with PKA consensus site versus no such site (Suppl. Fig. 3A). When comparing control and patient platelets, we found a moderate to strong reduction in altered proteins with PKA consensus site for patients P1,2,4,6 (Suppl. Fig. 3B). Again, platelets from P7 were deviant in showing an *in*crease in peptides with PKA consensus site. Taken together, these data indicated that the platelets from P1,2,4,6 and to a lesser extent from P3, responded different in iloprost-induced phosphorylation response, when compared to control platelets.

### Defining patterns of phosphoproteome changes by iloprost

To find specific patterns of phosphorylation changes, we listed those phosphopeptides with PKA consensus site that were most consistently changed in the patient platelets (see Table 4). Here, most pronounced phosphorylation defects were seen for patients P1,2,4,6 and lesser changes for P3. In contrast, multiple phosphopeptides were increased in patient P7, when compared to control platelets.

**Table 4:**
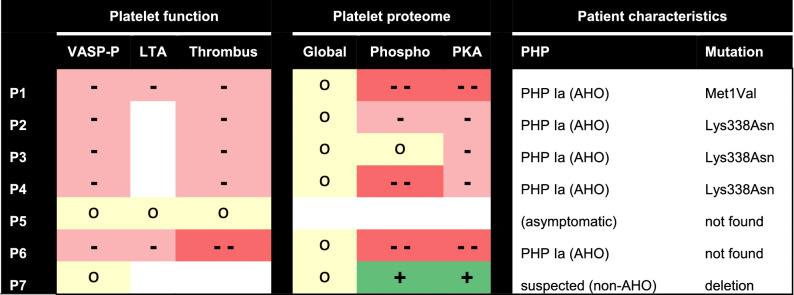
Overview of altered Gsα-dependent changes in platelet function and proteome of investigated patients.

Indicated are altered responses to iloprost or PGE_1_ per patient (P1-7), in comparison to control subjects on: (1) VASP Ser293 phosphorylation; (2) platelet aggregation by collagen-induced LTA; (3) microfluidic whole-blood thrombus formation; (4) global platelet proteome; (5) platelet phosphoproteome; (6) PKA phosphorylation sites. Colour code: red, decreased in comparison to controls; yellow, unchanged; green, increased; white, not determined. Last columns show clinical diagnosis regarding AHO and confirmed mutation in *GNAS.*

## Discussion

This paper reveals a set of dysfunctional Gsα-mediated responses in platelets from seven rare patients with established or suspected PHP Ia (Albright hereditary osteodystrophy, AHO), i.e. VASP phosphorylation, aggregation, and microfluidic thrombus formation. In all cases, the patient platelet were compared with day-control platelets from healthy control donors. These functional defects of platelets to Gsα stimuli (iloprost, PGE_1_) appeared to be accompanied by a consistent set of changes in the platelet protein phosphorylation pattern. Table 4 compares our findings for the five patients with confirmed PHP Ia (AHO), the asymptomatic family member P5 and the atypical patient P7.

Patients with PHP-related disorders present with characteristic phenotypic features, such as a short stature and brachydactyly, but diagnosis only by physical examination is notoriously difficult. Genetic screening is usually performed to confirm a maternally inherited mutation in the *GNAS* complex locus or to find a possible epigenetic defect^28^. If positive, the molecular characterisation PHP Ia is made, matching the clinical phenotype of AHO (OMIM: #103580). On the other hand, in the related disorder pseudopseudohypoparathyroidism (PPHP), mutations in the *GNAS* locus occur, which are usually paternally inherited and are designated as non-AHO. Molecular diagnosis of these syndromes is further complicated, as an AHO phenotype can also be accompanied by normal or hyper-activity of the Gsα protein^29^. Because of this complexity, platelet phenotyping for aberrant Gαs activity may assist in diagnosis.

In platelets, the Gsα-AC-PKA pathway, induced by endothelial prostacyclin (prostaglandin I_2_), is a main mechanism by which the vessel wall prevents activation^11,12^. Accordingly, iloprost or PGE_1_ addition to platelets—similarly to PKA inhibition—potently suppresses agonist-induced responses including Ca^2+^ fluxes, integrin activation, adhesion, secretion, aggregation and thrombus formation^15,30^. Since the early recognition that the platelet inactivation is controlled by PKA-dependent phosphorylation^31^, extensive analyses have been performed to characterize the iloprost-induced phosphoproteome^19,32^. The present paper provides a first report to extend this work to patients with suspected Gαs defects, in order to explore how this technology can aid in new biomarker finding and diagnosis^20,21^.

As indicated in overview Table 4, the investigated patients P1−4 (families I and II), with typical characteristics and maternal inheritance of a mutation in the *GNAS* locus, were all diagnosed as AHO. In family III, unlike the unaffected mother patient P5, patient P6 (child) with typical characteristics also obtained the ad-hoc diagnosis of AHO, although no mutation in *GNAS* was found (epigenetic analysis not yet performed). Patient P7 (family IV) was originally suspected for PHP Ia, as genetic screening revealed a heterozygous deletion mutation in *GNAS*. However, paternal inheritance is suspected, but not proven, which has led to a diagnosis of non-AHO. Markedly, our platelet phosphoproteome analysis is in accordance with a non-AHO phenotype.

Table 4 furthermore indicates overall consistency of impairments in the various Gsα-dependent platelet function tests, with respect to the blood samples from patients P1-4 and P6, when compared to day-control subjects. In the patient platelets, we noted an overall dysfunctional iloprost-induced VASP phosphorylation on Ser^239^, as a standard assay to check for Gsα-AC-PKA signalling^33,34^. Furthermore, for the same patients P1−4,6, we detected a dysfunctional response to PGE_1_ and iloprost, in terms of parameters of thrombus formation. Of note, due to the limited available blood samples, LTA could only be performed for two patients as a confirmation of this dysfunction. On the other hand, for patient P5 (unremarkably phenotype, mother of P6) and patient P7 (concluded as non-AHO), the performed platelet function tests were within normal ranges.

For platelets from six of the patients (P1−4,6–7) we measured the iloprost-induced phosphoproteome as an alternative way to detect abnormalities in the Gsα-AC-PKA pathway. Therefore, we aimed to quantify per patient: (1) the overall changes in iloprost-induced protein phosphorylation patterns in patients versus controls; (2) the changes that can be linked to PKA dependent phosphorylation; and (3) shortlist of consistently changed phosphoproteins.

In the five patients with established AHO (P1,2–4,6), we noticed a dose-dependent increase in upregulated, but not in downregulated phosphopeptides in response to iloprost (Table 5). Shortlisting these events to phosphopeptides with a PKA consensus site, we found that the platelets from four patients (P1,2,4,6) had a relatively large impairment in Gsα-AC-PKA dependent phosphorylation. In the platelets from P3, the aberrations were limited, while in the platelets from (non-AHO) P7 an increased, rather than decreased phosphorylation pattern was seen. This corroborated the findings on platelet functions. An unanswered question is why the platelets from P3 showed more subtle differences in Gsα-dependent phosphorylation than the platelets from family members P2,4 (all carrying the same *GNAS* mutation). This may be due to different thresholds in dose and time for Gsα-AC-PKA stimulation in the platelets from P3.

**Table 5:**
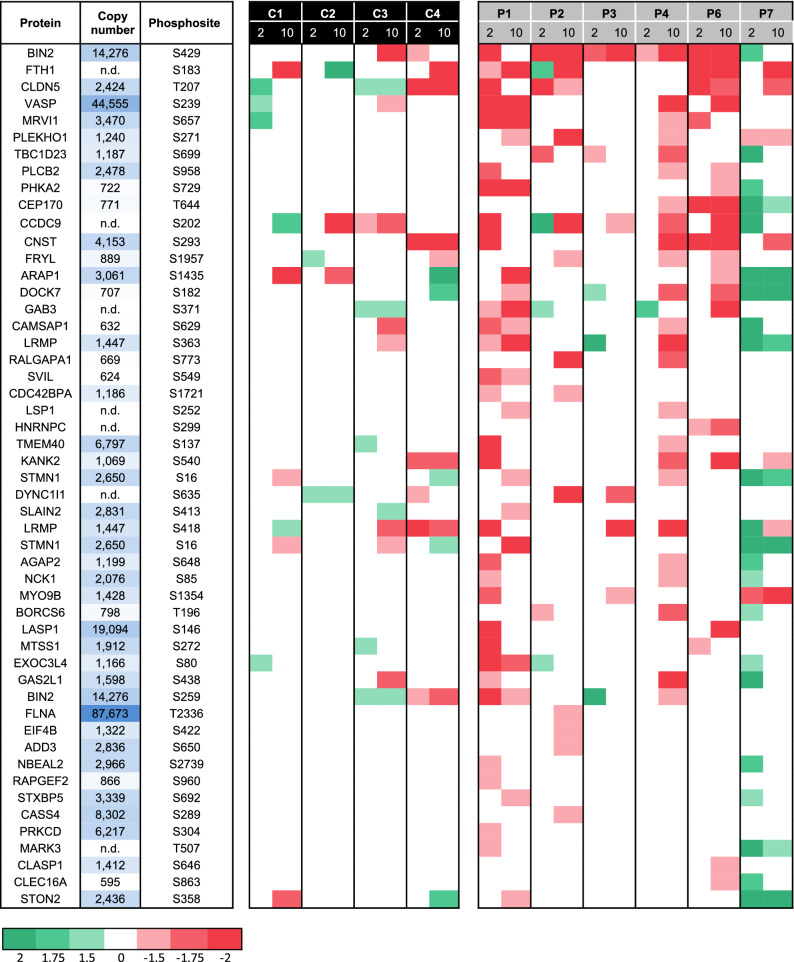
Shortlist of major PKA-dependent changes in iloprost-upregulated phosphoproteome in platelets from individual patients*.*

Identified iloprost-upregulated phosphopeptides with PKA consensus site and altered in platelets from indicated patients, in comparison to mean of controls (C’), with medium to strong effect size (Cohen’s *d* > 0.5). Colour bar: phosphopeptides that are fold decreased (red) or increased (green) compared to C’, for individual control subjects (C1-4) and patients (P1–4,6,7). Per phospho-site are indicated gene name and protein copy number^18^^.^

Reactome pathway analysis pointed to similar iloprost-induced changes in platelet functions, when evaluating the upregulated or the downregulated phosphoproteins. In either case, identified (sub)pathways were those of signal transduction, haemostasis, cell–cell interactions, and platelet activation. Jointly, this supports the presence of a coordinated mechanism of iloprost-induced platelet inhibition: directly mediated via PKA, and indirectly via a network of protein kinases as well as protein phosphatases^32^. Regarding the identified altered PKA-dependent phosphorylation events in confirmed AHO patients, several of the regulated proteins are of key importance for platelet signalling. These include the phospholipase C-β2 isoform (PLCB2); vasodilator-stimulated phosphoprotein (VASP) and the inositol-phosphate receptor regulator MRVI1^34^. In addition, phosphorylated proteins were listed that control platelet shape change, including a myosin isoform (MYO9B) and the tight junction protein claudin-5 (CLDN5). Differentially regulated also were the regulatory subunit RIIβ (PRKAR2B) of the PKA-II holoenzyme, as well as BIN2, a protein with unknown function, but previously recognised as one of the most strongly regulated proteins in PKA signalling^19^.

The current data are in support of the use of platelet phosphoproteomics in the diagnosis of AHO and related diseases, for instance by checking a panel of biomarker phosphorylation sites as in Table 5. However, it should also be mentioned that the mass spectrometric technique is expensive and requires trained personnel. On the other hand, the consistent, but still variable changes in the phosphoproteomes of multiple patients argues for a more extensive set of biomarker tests than only an anti-phospho VASP antibody.

Altogether, this work demonstrates an overall similarity in Gsα-AC-PKA mediated aberrations between functional responses and quantitative phosphoproteomics of platelets from patients with confirmed PHP Ia (AHO). The findings of aberrant upregulated as well as downregulated phosphopeptides in patients point to a change in the network of key protein kinases (starting from PKA) and phosphatases, which regulate multiple platelet functional properties.

## Materials and methods

### Materials

Iloprost was obtained from Bayer Schering Pharma (Leverkusen, Germany), and PGE_1_ from Fluka-Sigma Aldrich (Buchs, Switzerland). Horm type I collagen was purchased from Nycomed (Munich, Germany). Fluorescein isothiocyanate (FITC)-labelled antibody against phosphorylated vasodilator-stimulated phosphoprotein (P-VASP, phospho-Ser239) was from nanoTools (Teningen, Germany), Alexa Fluor (AF)647-labelled fibrinogen from Invitrogen Life Technologies (Bleiswijk, The Netherlands), FITC-labelled anti-CD62P mAb against P-selectin from Beckman Coulter (Marseille, France), and FITC-labelled PAC1 mAb against activated α_IIb_β_3_ integrin from Becton Dickinson (San Jose CA, USA). The membrane probe DiOC_6_ came from Anaspec (Reeuwijk, The Netherlands). Other materials were obtained from sources, as described before^35^.

### Patients and control subjects

Blood was obtained from healthy controls and indicated patients, after full informed consent and in accordance with the Declaration of Helsinki. Experiments were approved by the Ethics Committee of Maastricht University and Maastricht University Medical Centre. Blood samples from patients (P1–7) were freshly taken, and always analysed in parallel with blood samples of unrelated day-control subjects (C1–12). The numbering of individual patients and control subjects in this paper is unchanged for all assays. Platelet samples for proteomics assays were stored at an internal biobank, until assayed for this purpose on a later time point, such in accordance with the European data protection law.

Patients from four families were investigated with diagnosed or suspected PHP Ia (Table 1). based on elevated parathyroid hormone levels. All families showed typical symptoms associated with AHO, such as short stature and brachydactyly, while the degree of mental retardation was variable. Family I consisted of a mother (P1), who similarly to a son (not included), carried a heterozygous single nucleotide substitution in exon 1 (c.1A → G) of the *GNAS* complex locus, resulting in a truncated Gsα protein^36^. This mutation is known to be linked to an impaired expression of functional Gsα, thus reducing the Gsα bioactivity in blood cells^37^. Family II consisted of a mother (P2) and affected two sons (P3, P4), all of whom carried a heterozygous single nucleotide substitution in exon 338 (c.1G → C), encoding for a dysfunctional Gsα protein, with an estimated reduction of 46%, 49% and 51% in Gsα activity in the erythrocyte membranes^38^. Family III consisted of two related subjects, a mother (P5) with an unremarkable phenotype; and an affected daughter (P6) with PHP diagnosis, of whom no mutation in the *GNAS* locus could be detected (epigenetic imprinting analysis not performed). In family IV, patient P7 obtained a diagnosis, not differentiating between PHP and POH (progressive osseous heteroplasia), while genetic analysis revealed a heterozygous deletion in the *GNAS* locus (c.565_568del), linked to nonsense-mediated decay of mRNA (unpublished). Both parents did not allow examinations. In all 7 patients, blood platelet counts were within the normal range (Table 1). Patients and day control subjects had not used antiplatelet or anticoagulant medication for at least two weeks.

### Blood collection and platelet isolation

For whole blood perfusion experiments, blood samples were collected into 0.1 volume of saline containing d-phenylalanyl-prolyl-arginyl chloromethyl ketone (PPACK, 40 μM) and fragmin (40 U/mL)^39^. For light transmission aggregometry, blood was collected into 0.1 volume of 129 mM trisodium citrate. Platelet-rich plasma (PRP) was prepared by centrifuging blood samples at 240 g for 15 min. Platelet counts were determined with a thrombocounter (Coulter Electronics; Woerden, The Netherlands).

For measurements with washed platelets, including proteomics analyses, blood was collected into 0.1 volume of acid-citrate glucose solution (ACD, 52 mM citric acid, 80 mM trisodium citrate, 180 mM d-glucose)^35^. PRP was then obtained by centrifuging as above. After the addition of 0.066 volume of ACD, the platelets were pelleted by centrifugation at 870 g for 15 min. Pellets were resuspended in Hepes buffer pH 6.6 (136 mM NaCl, 2.7 mM KCl, 10 mM Hepes, 2 mM MgCl_2_ and 0.1% d-glucose), while carefully excluding any bottom layer of red cells. The suspended platelets were then transferred to a clean Eppendorf tube. After the addition of 0.066 volume ACD and apyrase (1 U/mL), collected platelets were washed by centrifugation at 2,000*g* for 5 min. Final resuspension then was in Hepes buffer pH 7.45 (136 mM NaCl, 2.7 mM KCl, 10 mM Hepes, 2 mM MgCl_2_, 0.1% d-glucose), once more by excluding any residual erythrocytes, followed by transfer to a clean Eppendorf tube. Purity of the final platelet preparations (1–2 × 10^8^/mL) for proteomics analyses was assessed by a thrombocounter and microscopic analysis. Contamination of platelets with red blood cells was < 1:15,000 and with leukocytes < 1:20,000.

### Flow cytometry

For analysis of VASP phosphorylation as a measure of Gsα-PKA activity, samples of purified platelet suspensions (2 × 10^8^/mL) were incubated with vehicle or iloprost (0.5–10 nM) for 1 min, after which reactions were stopped with 2% formaldehyde (in filtered phosphate-buffered saline with 0.2% bovine serum albumin). Fixed samples were centrifuged at 2,000*g* for 2 min, and pellets were washed twice with phosphate-buffered saline. The pelleted platelets were then resuspended in phosphate-buffered saline containing 0.1% saponin, to allow membrane permeabilization during 15 min. After addition of FITC-labelled anti-P-VASP mAb against phospho-Ser-239 (1:1,000), samples were incubated for 30 min, and analysed by flow cytometry (10,000 events/sample), using a BD Accuri C6 flow cytometer (San Jose CA, USA).

### Light transmission aggregometry

Aggregation of platelets in plasma (normalised to 3 × 10^8^ platelets/mL) was measured with an automated Chronolog aggregometer (Havertown PA, USA). The platelets were preincubated with vehicle (ethanol), PGE_1_ or iloprost for 4 min, and then activated with collagen (5 µg/mL) at 37 °C. Platelet aggregation rate was determined from the slopes of curves (% transmission change per min). In earlier functional experiments PGE_1_ was used, which was later replaced by iloprost because of the meanwhile published iloprost phosphoproteome^19^. Cyclic AMP measurements in washed platelets were performed, as before^40^.

### Microfluidic thrombus formation and platelet activation under flow

To measure whole blood thrombus formation, glass coverslips were coated with type I collagen and mounted into a Maastricht flow chamber, as described^41^. Samples of PPACK-anticoagulated blood, preincubated for 4 min with vehicle (ethanol), PGE_1_ (100 nM) or iloprost (5 or 10 nM), were perfused over the collagen surface at a wall shear rate of 1,000 s^-1^ for 4.0 min. Thrombi on coverslips were post-stained with FITC-labelled anti-P-selectin mAb (25 μg/mL)^25^. Brightfield differential interference contrast and confocal fluorescence images were taken from the collagen surface, as described^42^. Microscopic images were analysed for five variables (*V1-5*) using Image J (version 1.48 g, US NIH; Bethesda MD, USA)^25^. Deposited platelets (*V1*) and P-selectin staining (*V2*) were quantified as percentage of surface-area-coverage (%SAC); integrated feature size of aggregated platelets (*V3*) was expressed as μm^2^; thrombus multilayer score (*V4*) was scaled 0–3, depending on the presence of multilayered thrombi; and thrombus morphological score (*V5*) was scaled 0–5, ranging from no or single platelet adhesion to full thrombus formation^24^.

### Sample preparation for proteome analysis

Well-purified washed platelets (5 × 10^8^/mL) in Hepes buffer pH 7.45 were incubated with vehicle or iloprost (1–10 nM) for 1.0 min at 37 °C. Reactions were stopped by addition of 50% lysis buffer (50 mM Tris, 150 mM NaCl, 1% SDS, 1 tablet Roche PhosStop/7 mL buffer, pH 7.8, f.c.), and incubated on ice. Directly after lysis, the samples were snap-frozen, and then stored at − 80 °C until use. The following sample sets were simultaneously analysed (Suppl. Table 1). *Set I*: four samples from patient P1 and four samples from control subject C1 (i.e. resting, 1, 2 or 10 nM iloprost). *Set II*: four samples from patient P7 and four samples from control subject C4 (resting, 2, 5 or 10 nM iloprost). *Set III*: three samples for patient P2, three samples for patient P6, three samples for control subject C2 (resting, 2 or 10 nM iloprost), and a calibration sample. *Set IV*: three samples for patient P3, three samples for patient P4, three samples for control subject C3, (resting, 2 or 10 nM iloprost) and a calibration sample. In parallel, platelet samples were analysed for VASP-P determination. Platelets from asymptomatic patient P5 were not available for this analysis.

### Protein digestion and stable isotope labelling for proteomic analysis

Sample preparation, proteolytic digestion and iTRAQ/TMT labelling were based on previously described methods^18,27,43,44^. The TMT labelling was performed according to the manufacturer's instructions. Quality control of all samples was as before^19^. Lysed samples of the purified platelets (5 × 10^8^/mL) were diluted to the same protein concentration (checked with a bicinchoninic acid protein assay kit; Pierce, Thermo-Fisher Scientific, Bremen, Germany). Cysteines were reduced (30 min, 56 °C) and free sulfhydryl groups were alkylated (30 min, room temperature, in darkness) with 10 mM dithiothreitol and 30 mM iodoacetamide, respectively. Samples of sets I–IV were handled in equivalent manners, as described below.

For sets I and II, aliquots containing 100 µg of protein were diluted tenfold with ice-cold ethanol and incubated for 1 h at − 40 °C. The mixtures were centrifuged for 30 min at 4 °C and 18,000*g*, and supernatants were carefully removed. The precipitates were washed using 50 µL of ice-cold acetone, followed by a 15 min centrifugation. This step was repeated once. Precipitated proteins were re-solubilised into 6 M guanidine hydrochloride, and digested in-solution with trypsin (Sequence grade modified, Promega, Madison WI, USA) at a 1:20 enzyme:protein ratio, with a final concentration of 0.2 M guanidine hydrochloride, 2 mM CaCl_2_ and 50 mM triethylammonium bicarbonate (14 h, 37 °C). Digestion controls were performed, using a monolithic-RP HPLC system, as before^45^. Digests were individually labelled with iTRAQ 8-plex labels (113–119, 121). After drying in vacuum, the samples were dissolved in iTRAQ 8-plex dissolution buffer (AB Sciex; Dreieich, Germany), and labelled according to the manufacturer’s protocol. Samples per set were pooled at 1:1 ratios, were desalted by C_18_ solid phase extraction (SPEC C_18_ AR, 4 mg bed; Agilent Technologies, Brussels, Belgium) and dried under vacuum.

For sets III and IV, 150 µg protein per sample was loaded onto a 30 kDa molecular weight cut off spin filter to perform filter-aided sample preparation, as described elsewhere^46,47^, with slight modifications. Proteins were digested (14 h, overnight) in 50 mM triethylammonium bicarbonate, 0.2 M guanidine hydrochloride, 2 mM CaCl_2_, pH 8.5 with trypsin (w/w 1:25, sequencing grade, Promega, USA)^19^. The peptides were collected by centrifugation at 14,000*g* for 20 min. To increase peptide yield, filters were additionally washed with 50 µL 50 mM triethylammonium bicarbonate, and subsequently with 50 µL water. Digestion performance and peptide yield was controlled, as previous^48^. Digests were individually labelled with TMT 10-plex labels (126, 127N, 127C, 128N, 128C, 129N, 129C, 130N, 130C, 131, from Thermo Scientific). Equal peptide amounts of vacuum dried samples were reconstituted in 100 µL 100 mM triethylammonium bicarbonate, and labelled with 0.8 mg reagent according to the manufacturer’s protocol. Samples were then pooled at 1:1 ratios, and further treated similar to sets I + II.

The multiplexed iTRAQ or TMT pools were used to quantify the global proteomes and the phosphoproteomes of the individual samples. For global proteome analysis, 10% (iTRAQ) or 3.5% (TMT) of a pooled mixture was pre-fractioned on a U3000 HPLC (Thermo Scientific) by high pH reversed-phase chromatography (C_18_ column; BioBasic-18, 0.5 mm ID × 15 cm, 5 µm particle size, 300 Å pore size, Thermo Scientific) using a linear gradient. For iTRAQ pools, this gradient was ranging from 3–50% solvent *B* (mobile phase *A*: 10 mM ammonium acetate, pH 6.0, *B*: 10 mM ammonium acetate, 84% acetonitrile, pH 6.0, 75 min). For TMT pools, a linear gradient ranged from 3–50% solvent *B* (mobile phase *A*: 10 mM ammonium formate pH 8.0, *B*: 10 mM ammonium formate, 84% acetonitrile, pH 8.0, 75 min) to obtain 20 concatenated fractions for LC–MS analysis.

For phosphopeptide analysis, the remaining of the pooled iTRAQ or TMT samples were subjected to a TiO_2_-based phosphopeptide enrichment protocol, as described elsewhere with slight modifications^43^. Briefly, samples were resuspended in TiO_2_ loading buffer (80% acetonitrile, 5% trifluoroacetic acid, 1 M glycolic acid), and incubated twice with TiO_2_ beads for 10 min. Incubations first had a peptide to bead ratio of 1:6, and then a ratio of 1:3. For set III or IV, an additional incubation at 1:1.5 ratio was performed. Subsequently, the beads of all incubation steps were combined in one Eppendorf tube, and washed and eluted, as previously^49^. In short, 80% acetonitrile, 1% trifluoroacetic acid was used for washing step 1, and 10% acetonitrile, 0.1% trifluoroacetic acid for washing step 2. The phosphopeptides were eluted by incubation with 1% NH_4_OH for 10 min. The eluates were acidified using formic acid (pH < 2). To obtain better phosphopeptide recovery, the enrichment procedure was repeated once with a slight variation, i.e. a loading buffer of 70% acetonitrile, 2% trifluoroacetic acid, and a washing buffer of 50% acetonitrile, 0.1% trifluoroacetic acid. Phosphopeptides were eluted as described above and then acidified.

The acidified phosphopeptides were desalted using Oligo R3 micro-columns^50^, and fractionated on a U3000 RSLC system in hydrophilic interaction liquid chromatography (HILIC) mode (Polar phase TSKgel Amide-80; 150 µm ID × 15 cm length; 5 µm particle size; 80 Å pore size, Tosoh Bioscience, Tessenderlo, Belgium), using a binary gradient ranging from 10–35% solvent *B* (solvent *A*: 98% acetonitrile, 0.1% trifluoroacetic acid; solvent *B*: 0.1% trifluoroacetic acid) in 40 min (flow rate: 4 µL/min). A total of 9 fractions per set were collected for subsequent LC–MS analysis.

### Global proteome and phosphoproteome analysis by iTRAQ or TMT labelling

Sets I and II (*in italic sets III and IV, if different*): RP and HILIC fractions were individually analysed by nano-LC/MS–MS, using a Ultimate 3,000 RSLC-nano system online-coupled to a Q-Exactive Plus (iTRAQ) or a Q-Exactive HF (TMT) mass spectrometer (both Thermo Scientific). Individual fractions were loaded onto a trap column (Acclaim PepMap100 C_18_ trap column; 100 μm × 2 cm) with 0.1% trifluoroacetic acid; flow rate: 20 µL/min. This was followed by separation of peptides on the main column (PepMap100 C_18_; 75 μm × 50 cm), using a binary gradient ranging from 3–42% (*3–35%*) solvent *B* [84% acetonitrile, 0.1% formic acid] in 145 min (*60* min). In the Q-Exactive, survey scans were acquired at resolution of 70,000 (60,000) using an automatic gain control (AGC) target value of 3 × 10^6^ (1 × 10^6^). MS/MS spectra of the top 15 most intense ions were acquired with a resolution of 17,500 (60,000), an isolation width of 2.0 (*0.8*) *m*/*z*, a normalised collision energy of 35% (*33%*), an AGC target value of 1 (*2*) × 10^5^ ions, a maximum injection time of 250 (*200*) ms and a dynamic exclusion of 12 *(30) s* with and underfill ratio of 10%. The first fixed mass was set to 105 (100) *m*/*z*. In order to compensate for the iTRAQ-induced increase of peptide charge states, reaction tubes with 10% ammonium water were placed in front of the ion source as described elsewhere^51^. For TMT samples, to compensate for a higher complexity of the global proteome fractions, the isolation width was reduced to 0.4 m*/z* to reduce the potential precursor co-isolation.

Raw data were processed with Proteome Discoverer 1.4 (Thermo-Fisher Scientific). Data were searched against the Uniprot human database (August 2012; 20,232 target sequences) using Mascot and Sequest with the following settings: (1) trypsin as enzyme allowing two missed cleavages, (2) iTRAQ 8-plex (*TMT 10-plex*) at N-termini and lysines of + 304.2053 (+ 229.163) Da and carbamidomethylation of Cys + 57.0214 Da as fixed modifications, (3) oxidation of Met + 15.9949 Da as variable modification, (4) mass tolerances of 10 ppm and to 0.02 Da for MS and MS/MS, respectively. For HILIC fractions, phosphorylation of Ser/Thr/Tyr (+ 79.9663 Da) was selected as additional variable modification. False discovery rate (FDR) estimation on the level of peptide spectrum matches (PSM) was performed using the peptide validator node, filtering for high confidence 1% FDR. The reporter ion quantifier node was used for iTRAQ (*TMT*) reporter quantification.

For global proteome quantification (RP fractions) only unique proteins quantified with at least 2 unique peptides were considered. For phosphoproteome quantification (HILIC fractions), phosphorylation site localisation was determined using phospho-RS^52^, and only phosphopeptides with phospho-RS site probabilities > 90% were considered as confident. Sorting and evaluation of the exported data as well as calculations were done in Microsoft Excel.

### Data analysis of platelet global proteomes and phosphoproteomes

Sets I + II (sets III + IV): as proteome discoverer only provided 7 (9) ratios for the 8 (10) samples, an artificial 113/113 (126/126) ratio was created and set to 1.0 per protein and all ratios were log2 transformed. Per channel, the median ratio over all proteins was calculated, from which the median of all eight (ten) values was determined to define normalisation factors per iTRAQ *(TMT)* channel. These factors compensated for systematic errors (i.e.*,* unequal sample amounts derived from pipetting errors or inaccurate protein determination results) and allowed to obtain normalised ratios per protein. The normalised ratios were divided by the median (log2 transformed) over all eight *(ten)* values to obtain scaled normalised abundance values (NAVs) for all proteins and channels. The NAVs (log2 transformed) allowed determination of inter-sample ratios for platelets from the patients and control subjects for each condition.

Inter-individual variation for the global proteome was estimated by separate analysis of the samples from all subjects (combined sets) in comparison to averaged control values (C1–4). Differences from average control values were thresholded based on altered ratios log2 transformed (≥ 25% up or downregulation: range of − 0.322 to + 0.322).

For determination of confident phosphorylation sites at the peptide level, a ready-to-use Excel macro provided by Mechtler lab (https://ms.imp.ac.at/?goto=phosphors) was used. Data were normalised as described above. NAVs were calculated per phospho-site, as described for the global proteome. Average NAVs were calculated per iTRAQ or TMT channel, after grouping per phosphopeptide sequence, phosphorylation site and protein. Phosphopeptides present in ≥ 3 control samples were used to assess average control values, after normalisation per data set, and were log2 transformed. Treatment effects were estimated based on an arbitrary cut-off of ≥ 25% up- or downregulation (log2 range of − 0.322 to + 0.322). Thresholds for relevant changes in patient samples were more strictly set at 2 × (− 0.322 to + 0.322); comparisons were made to mean control NAVs, for up- or downregulation per phosphopeptide.

### Reference values of phosphoproteomes and determination of PKA phosphorylation sites

Phosphorylation data were compared with a reference dataset of iloprost-induced changes in protein phosphorylation of healthy control platelets (2,700 phosphopeptides, of which 299 regulated by iloprost), as published^19,32^. Iloprost-regulated phosphopeptides were defined as those responsive to 1 min treatment with 2 or 10 nM iloprost. A three-point scale was used (1 = upregulated, 0 = unchanged, − 1 = downregulated). Consensus sites for PKA-induced phosphorylation were as before^19^, using the GPS2.1 algorithm for kinase consensus sequence prediction^53^. Classification of proteins was as before^27^.

### Reactome pathway analysis

Lists of protein identifiers (gene names, Uniprot) were introduced in the Reactome pathway database (reactome.org), and analysed on biological pathways with n > 4 entities and false discovery rates of > 0.82. Lists contained phosphorylated proteins regulated by 2 and/or 10 nM iloprost: 196 upregulated and 159 downregulated. Weight factors were included, presenting the numbers of regulated phosphorylation sites per protein.

### Experimental design and rationale

Inclusion of the quite rare patients with suspected PHP Ia (expected prevalence of 1/250,000, but underdiagnosed) occurred over the years 2014–2017. As required for diagnostic platelet function analyses, blood samples from the 8 patients were directly compared with samples from a healthy day-control subject (in total 12 controls).The common rationale in diagnostic laboratories is that the (isolated) platelets from all healthy subjects make up a normal pool (with defined normal ranges of test outcomes) that, however, needs to be validated day-by-day to check for the quality of blood drawing and sample preparation. At a first visit, blood from patients and day controls was analysed for platelet functions with Gsα-stimulating agents (platelet aggregation, microfluidics, flow cytometry). In addition, platelet samples were made for proteomics analysis (both global proteome and phospho-proteome). If possible and required, at a second visit, blood from patients (e.g., children) and controls was used for additional functional assays. Note that flow cytometry of VASP phosphorylation was performed with all samples. The total number of included controls was 12.

Given the earlier established high stability of the platelet proteome and phosphoproteome among healthy subjects^18,19^ and to retain an affordable work-flow, for the proteomic assays, for omics analyses we compared patient samples with the means of respective day-control samples. Sets I-II of platelet and control samples, obtained in 2014–2015, were analysed in 8-plex using iTRAQ labelling. Set III-IV samples, obtained in 2017, were analysed in 10-plex using TMT labelling.

### Statistics

Functional and proteomics data of control subjects are represented as medians ± interquartile ranges; data from patients are indicated individually. Differences of individual patients were considered to be statistically significant, when outside the normal ranges (defined by 95% confidence intervals). Effect sizes were calculated from the Cohen’s *d*, i.e. (M_1_ − M_2_)/SD_M1+M2_, in which M_1_ refers for means of patients and M_2_ to means of controls). For medium effect size, we used a *d* ≥ 0.5. Cohen's *d* calculates a standardised difference between both groups that are not affected by sample size. Heatmaps were created using the R package version 3.5.1. Unsupervised hierarchical clustering of datasets from patients and controls was also performed in R.

## Supplementary information

Datafile S1

Datafile S2

Supplementary information
