# Supplementary material for: Impaired iloprost-induced platelet inhibition and phosphoproteome changes in patients with confirmed pseudohypoparathyroidism type Ia, linked to genetic mutations in GNAS
Source: Sci Rep. 2020 Jul 9;10:11389. doi: 10.1038/s41598-020-68379-3 (PMC7347634; doi:10.1038/s41598-020-68379-3)
Supplement: Supplementary file 3 — Supplementary information [file 41598_2020_68379_MOESM3_ESM.docx]

***Scientific Reports*** Revised submission ID 85e04d1f-b5ee-4901-b483-c610c3ef7a56

**Impaired iloprost-induced platelet inhibition and phosphoproteome changes in patients with confirmed pseudohypoparathyroidism type Ia, linked to genetic mutations in GNAS**

Frauke Swieringa, Fiorella A. Solari, Oliver Pagel, Florian Beck, Marion A.H. Feijge, Kerstin Jurk, Irene M.L.W. Körver-Keularts, Nadine J.A. Mattheij, Jingnan Huang, Jörg Faber, Joachim Pohlenz, Alexandra Russo, Connie T.R.M. Stumpel, Dirk E. Schrander, Barbara Zieger, Paola E.J. van der Meijden, René P. Zahedi, Albert Sickmann, Johan W.M. Heemskerk

Correspondence: Johan W. M. Heemskerk, Department of Biochemistry (CARIM), Maastricht University, PO Box 616, 6200 MD Maastricht, The Netherlands. Tel: +31-43-3881674, fax: +31-43-3884159; e-mail: jwm.heemskerk@maastrichtuniversity.nl

**Supplemental figures and tables**


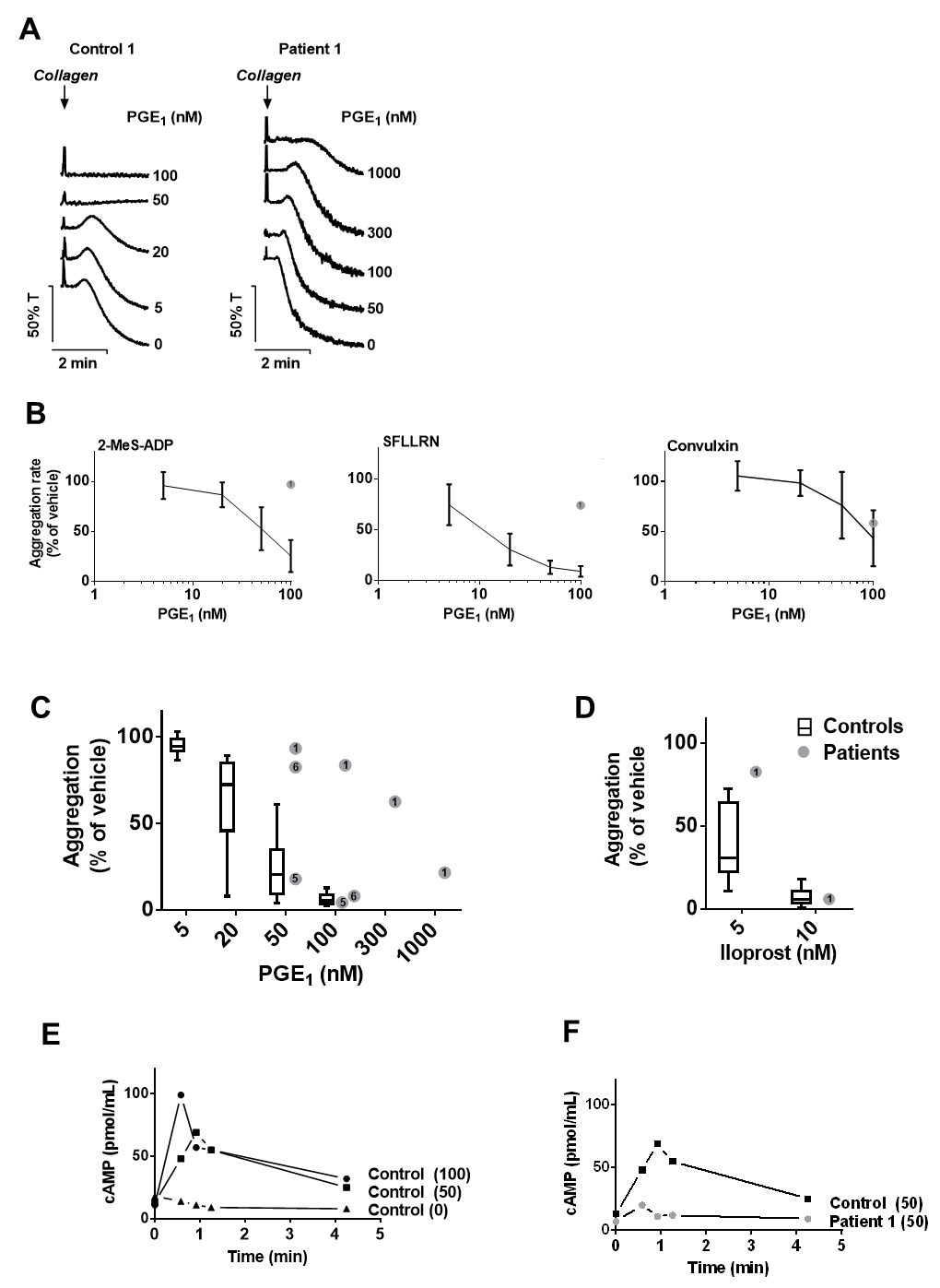


**Suppl. Figure 1.** *Impaired Gsα-mediated inhibition of aggregation and cAMP rise in platelets from patients with PHP Ia***.** Platelet-rich plasma was preincubated with vehicle or indicated concentration of PGE_1_ or iloprost (in nM) for 4 min. (**A**) Representative aggregation traces of platelets from day control subject C1 and patient P1. (**B**) Effect for of PGE_1_ on aggregation rate with Me-ADP (5 μM), SFLLRN (15 µM) or convulxin (10 ng/mL). Data from C1-10 and P1. (**C,D**) Dose-dependent effect of PGE_1_ (**C**) or iloprost (**D**) on platelet aggregation rate for controls C1-10 and patients P1,5,6. Box plots indicate medians ± interquartile ranges (whiskers represent 2.5-97.5^th^ percentiles, *n* = 10). (**E, F**) Transient effect of PGE_1_ (nM) on cAMP level in washed platelets from control C1 (**E**) and patient P1 (**F**).


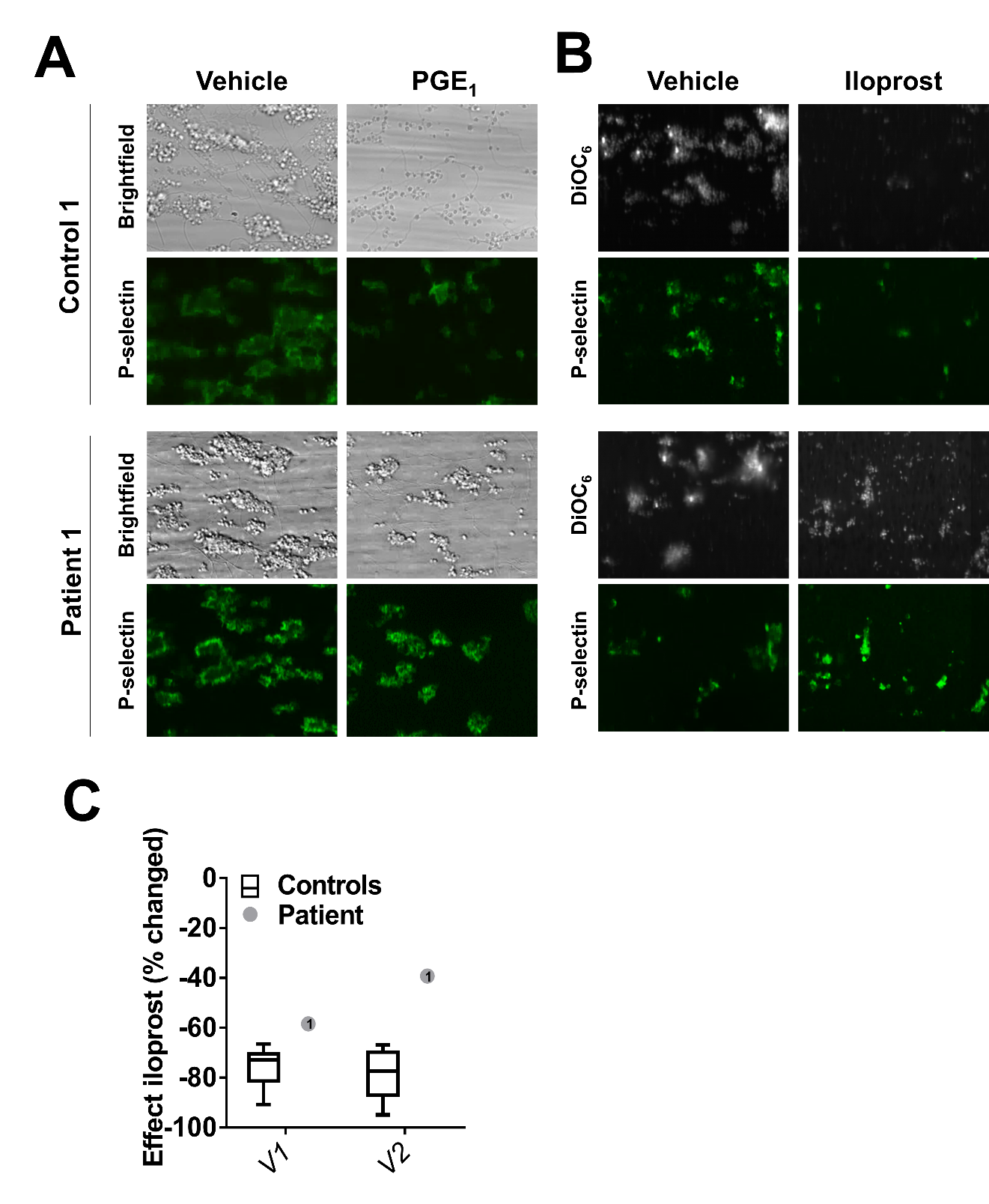


**Suppl. Figure 2.** *Impaired Gsα-mediated inhibition of thrombus formation in patient P1.* Blood samples from control subject (C1) and patient (P1) were perfused over collagen, and brightfield or fluorescence microscopic images were captured, as in Fig. 2. Samples were preincubated with vehicle, PGE_1_ (100 nM) or iloprost (10 nM), as indicated. (**A**, **B**) Representative brightfield and fluorescence images (FITC anti-P-selectin mAb, DiOC_6_-labelled platelets), illustrating effects of PGE_1_ and iloprost. (**C**) Quantitative effect of iloprost on parameters *V1* (platelet adhesion) and *V2* (P-selectin expression) for all control subjects (C1-8) and patient (P1). Medians ± interquartile ranges (whiskers represent 2.5-97.5th percentiles, *n* = 8).


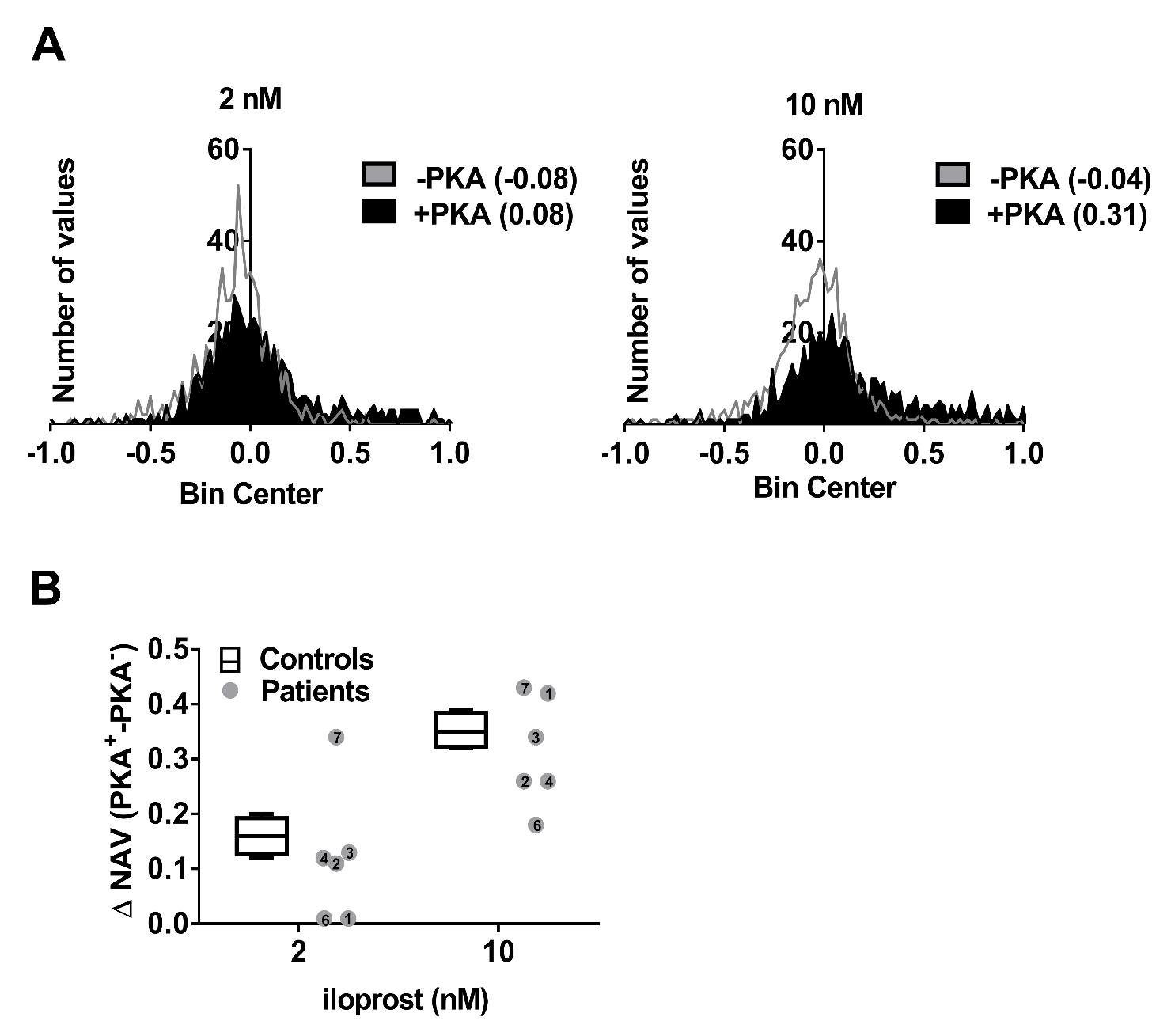


**Suppl. Figure 3**. *Altered iloprost effect on PKA-mediated phosphorylation in patient platelets.* (**A**) Histograms of normalised abundance values (NAVs) for all phosphopeptides identified in control platelets (C', C1-4), illustrating the effects of 2 or 10 nM iloprost. Phosphopeptides were separated according to the absence (PKA**^-^**) or presence (PKA**^+^**) of PKA consensus site. (**B**) Mean difference of NAVs for PKA**^+^** and PKA**^-^** phosphopeptides, shown for platelets from controls (medians ± interquartile ranges) and from patients P1-4,6,7.

**Suppl. Table 1.** *Sample sets for patients and corresponding day controls used for (phospho)proteome analysis.* Sets of platelet samples from controls (C) and patients (P) simultaneously assessed. Indicated samples were combined per set I-IV for iTRAQ labelling (8-plex) or TMT labelling (10-plex). Platelets from P5 were not available for this analysis.


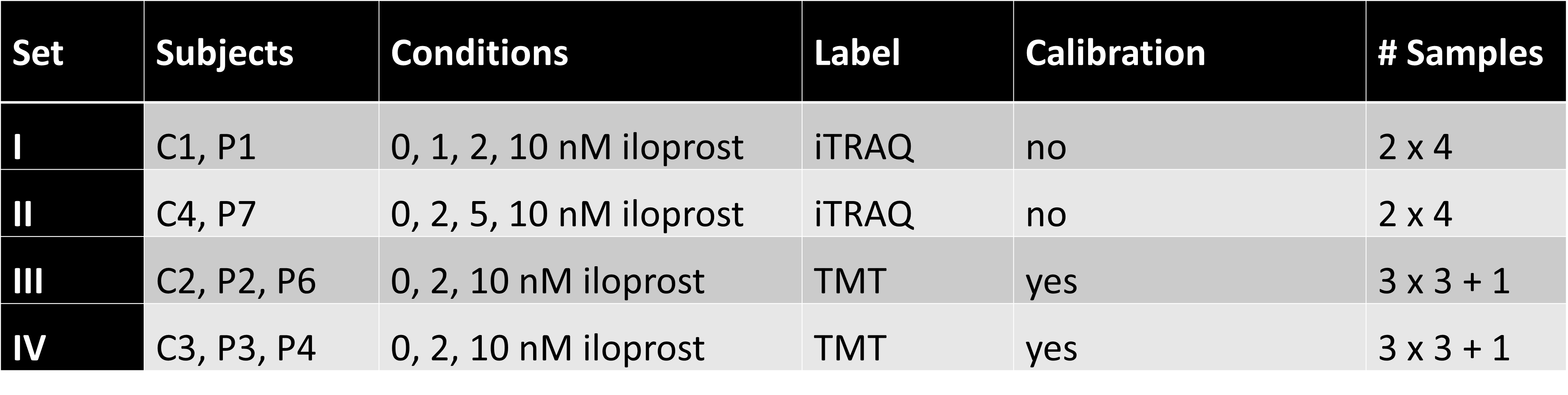


**Suppl. Table 2.** *Reactome pathway analysis of 196 iloprost-upregulated (up) and 159 iloprost-downregulated (down) phosphoproteins (entities).* Indicated are Reactome pathway identifiers; pathway names; entities identified as up- or downregulated; total numbers of entities in pathway; numbers of identified interactors per pathway; mean false discovery rate (FDR) based on null hypothesis of unchanged; numbers of reactions in Reactome; and mean numbers of phosphosites (weight) per pathway. Shown are top 62 pathways (Σ entities + interactors) with FDR<0.16. Pathways in bold are relevant for platelet activation and inhibition.

| **Pathway identifier** | **Pathway name** | **#Entities found up** | **#Entities found down** | **#Entities total** | **#Interactors found up** | **#Interactors found down** | **Entities FDR u/d** | **#Reactions found up** | **#Reactions found down** | **#Weight u/d** |  |
| --- | --- | --- | --- | --- | --- | --- | --- | --- | --- | --- | --- |
| R-HSA-162582 | **Signal Transduction** | 51 | 44 | 3303 | 113 | 83 | 0.01 | 652 | 453 | 1.4 |  |
| R-HSA-168256 | Immune System | 46 | 36 | 2822 | 60 | 79 | 0.01 | 381 | 305 | 1.3 |  |
| R-HSA-1280215 | **Cytokine Signaling in Immune system** | 19 | 17 | 1261 | 67 | 20 | 0.01 | 192 | 23 | 1.3 |  |
| R-HSA-1266738 | Developmental Biology | 17 | 21 | 1207 | 47 | 38 | 0.01 | 132 | 142 | 1.3 |  |
| R-HSA-1643685 | Disease | 11 | 14 | 1552 | 52 | 46 | 0.01 | 148 | 153 | 1.3 |  |
| R-HSA-392499 | Metabolism of proteins | 10 | 16 | 2354 | 47 | 39 | 0.01 | 95 | 103 | 1.2 |  |
| R-HSA-449147 | **Signaling by Interleukins** | 11 | 8 | 639 | 58 | 29 | 0.01 | 134 | 59 | 1.3 |  |
| R-HSA-168249 | Innate Immune System | 23 | 19 | 1328 | 28 | 33 | 0.01 | 130 | 151 | 1.2 |  |
| R-HSA-74160 | Gene expression (Transcription) | 7 | 3 | 1822 | 51 | 38 | 0.01 | 110 | 85 | 1.4 |  |
| R-HSA-73857 | RNA Polymerase II Transcription | 7 | 3 | 1664 | 51 | 37 | 0.01 | 107 | 83 | 1.4 |  |
| R-HSA-9006934 | **Signaling by Receptor Tyrosine Kinases** | 16 | 8 | 553 | 39 | 34 | 0.01 | 281 | 135 | 1.4 |  |
| R-HSA-422475 | Axon guidance | 16 | 19 | 584 | 36 | 25 | 0.11 | 111 | 101 | 1.3 |  |
| R-HSA-212436 | Generic Transcription Pathway | 7 | 3 | 1525 | 50 | 35 | 0.01 | 101 | 80 | 1.4 |  |
| R-HSA-109582 | **Hemostasis** | 22 | 2 | 821 | 34 | 33 | 0.11 | 82 | 78 | 1.3 |  |
| R-HSA-1430728 | Metabolism | 24 | 4 | 3636 | 30 | 25 | 0.01 | 102 | 86 | 1.3 |  |
| R-HSA-597592 | Post-translational protein modification | 7 | 9 | 1592 | 35 | 32 | 0.01 | 71 | 63 | 1.1 |  |
| R-HSA-372790 | **Signaling by GPCR** | 13 | 13 | 1484 | 30 | 26 | 0.01 | 66 | 56 | 1.3 |  |
| R-HSA-5653656 | **Vesicle-mediated transport** | 18 | 20 | 824 | 19 | 25 | 0.13 | 83 | 82 | 1.2 |  |
| R-HSA-1280218 | Adaptive Immune System | 14 | 11 | 999 | 28 | 27 | 0.01 | 64 | 65 | 1.3 |  |
| R-HSA-199991 | **Membrane Trafficking** | 17 | 19 | 665 | 19 | 25 | 0.13 | 79 | 80 | 1.2 |  |
| R-HSA-388396 | **GPCR downstream signalling** | 13 | 13 | 1358 | 26 | 21 | 0.01 | 59 | 47 | 1.2 |  |
| R-HSA-76002 | **Platelet signaling and aggregation** | 10 | 10 | 293 | 25 | 22 | 0.15 | 46 | 47 | 1.4 |  |
| R-HSA-5663202 | **Diseases of signal transduction** | 10 | 8 | 484 | 25 | 19 | 0.02 | 92 | 82 | 1.4 |  |
| R-HSA-73887 | Death Receptor Signalling | 6 | 4 | 157 | 20 | 20 | 0.01 | 23 | 23 | 1.2 |  |
| R-HSA-556833 | **Metabolism of lipids** | 8 | 2 | 1445 | 19 | 19 | 0.01 | 56 | 65 | 1.3 |  |
| R-HSA-5663205 | Infectious disease | 1 | 7 | 540 | 21 | 17 | 0.01 | 40 | 54 | 1.1 |  |
| R-HSA-2682334 | **EPH-Ephrin signaling** | 4 | 7 | 101 | 19 | 15 | 0.11 | 46 | 44 | 1.3 |  |
| R-HSA-4420097 | VEGFA-VEGFR2 Pathway | 5 | 4 | 126 | 18 | 17 | 0.10 | 48 | 28 | 1.2 |  |
| R-HSA-9006925 | **Intracellular signaling by second messengers** | 8 | 3 | 362 | 14 | 17 | 0.01 | 39 | 17 | 1.3 |  |
| R-HSA-354192 | **Integrin alphaIIb beta3 signaling** | 5 | 2 | 39 | 14 | 19 | 0.15 | 21 | 21 | 1.4 |  |
| R-HSA-9006921 | **Integrin signaling** | 5 | 2 | 39 | 19 | 14 | 0.15 | 21 | 21 | 1.4 |  |
| R-HSA-1640170 | Cell Cycle | 8 | 4 | 682 | 19 | 8 | 0.01 | 82 | 68 | 1.2 |  |
| R-HSA-373752 | Netrin-1 signaling | 4 | 3 | 59 | 14 | 17 | 0.12 | 16 | 10 | 1.4 |  |
| R-HSA-166520 | Signaling by NTRKs | 5 | 0 | 118 | 15 | 16 | 0.02 | 40 | 14 | 1.3 |  |
| R-HSA-109581 | **Apoptosis** | 4 | 5 | 189 | 15 | 11 | 0.02 | 22 | 18 | 1.2 |  |
| R-HSA-8953897 | Cellular responses to external stimuli | 9 | 7 | 586 | 15 | 4 | 0.01 | 51 | 25 | 1.2 |  |
| R-HSA-5357801 | **Programmed Cell Death** | 4 | 5 | 197 | 15 | 11 | 0.02 | 22 | 18 | 1.1 |  |
| R-HSA-418594 | **G alpha (i) signalling events** | 5 | 5 | 566 | 14 | 10 | 0.01 | 26 | 16 | 1.4 |  |
| R-HSA-3700989 | Transcriptional Regulation by TP53 | 4 | 1 | 486 | 19 | 10 | 0.01 | 34 | 15 | 1.3 |  |
| R-HSA-382551 | Transport of small molecules | 9 | 7 | 965 | 10 | 8 | 0.01 | 21 | 25 | 1.2 |  |
| R-HSA-202733 | **Cell surface interactions at the vascular wall** | 5 | 5 | 257 | 14 | 8 | 0.13 | 21 | 11 | 1.2 |  |
| R-HSA-162906 | HIV Infection | 0 | 5 | 262 | 16 | 10 | 0.01 | 20 | 31 | 1.1 |  |
| R-HSA-2454202 | Fc epsilon receptor (FCERI) signaling | 4 | 4 | 235 | 12 | 10 | 0.05 | 31 | 26 | 1.3 |  |
| R-HSA-112316 | Neuronal System | 6 | 5 | 499 | 12 | 6 | 0.01 | 24 | 28 | 1.3 |  |
| R-HSA-75153 | **Apoptotic execution phase** | 4 | 4 | 54 | 12 | 8 | 0.15 | 13 | 10 | 1.1 |  |
| R-HSA-69278 | Cell Cycle, Mitotic | 8 | 4 | 570 | 12 | 4 | 0.01 | 69 | 53 | 1.2 |  |
| R-HSA-186763 | **Downstream signal transduction** | 4 | 0 | 37 | 14 | 10 | 0.12 | 12 | 5 | 1.4 |  |
| R-HSA-983231 | **Megakaryocytes and platelet production** | 8 | 4 | 194 | 6 | 4 | 0.15 | 11 | 8 | 1.4 |  |
| R-HSA-381119 | Unfolded Protein Response (UPR) | 2 | 5 | 155 | 11 | 4 | 0.01 | 9 | 11 | 1.7 |  |
| R-HSA-1483249 | **Inositol phosphate metabolism** | 5 | 9 | 90 | 4 | 3 | 0.15 | 6 | 3 | 1.7 |  |
| R-HSA-446203 | Asparagine N-linked glycosylation | 3 | 5 | 421 | 4 | 8 | 0.05 | 16 | 22 | 1.2 |  |
| R-HSA-500792 | **GPCR ligand binding** | 1 | 4 | 652 | 6 | 5 | 0.01 | 7 | 9 | 1.3 |  |
| R-HSA-397014 | Muscle contraction | 3 | 6 | 256 | 6 | 0 | 0.15 | 10 | 12 | 1.6 |  |
| R-HSA-948021 | Transport to the Golgi and modification | 3 | 5 | 219 | 4 | 3 | 0.12 | 16 | 17 | 1.2 |  |
| R-HSA-2559583 | Cellular Senescence | 4 | 3 | 198 | 6 | 1 | 0.01 | 9 | 7 | 1.3 |  |
| R-HSA-2428924 | **IGF1R signaling cascade** | 4 | 0 | 72 | 5 | 5 | 0.13 | 11 | 3 | 1.8 |  |
| R-HSA-2428928 | **IRS-related events triggered by IGF1R** | 4 | 0 | 69 | 5 | 5 | 0.13 | 9 | 3 | 1.8 |  |
| R-HSA-68886 | M Phase | 6 | 4 | 390 | 3 | 1 | 0.15 | 25 | 20 | 1.3 |  |
| R-HSA-373755 | **Semaphorin interactions** | 1 | 6 | 70 | 2 | 5 | 0.11 | 7 | 11 | 1.6 |  |
| R-HSA-2404192 | **Signaling by Type 1 IGF1R** | 4 | 0 | 73 | 5 | 5 | 0.13 | 11 | 3 | 1.8 |  |
| R-HSA-71387 | Metabolism of carbohydrates | 9 | 0 | 456 | 1 | 1 | 0.08 | 13 | 1 | 1.0 |  |
| R-HSA-418346 | **Platelet homeostasis** | 1 | 4 | 123 | 2 | 0 | 0.03 | 5 | 5 | 1.5 |  |

**Suppl. Table 3.** *Gene Ontology resource analysis (geneontology.org) of 196 iloprost-upregulated phosphoproteins according to GO biological processes.* Shown are main discriminative processes, ranked according to numbers of items covered, fold enrichment and false discovery rates (FDR).

**GO biological process (main) Items Fold enrichment FDR**

Response to stimulus 173 1.37 1.98E-5

Signal transduction 127 1.69 5.17E-8

Regulation of signalling 104 1.95 2.00E-9

Cytoskeleton organization 68 4.18 1.34E-19

Cell surface receptor signalling 63 1.75 8.40E-4

Regulation of phosphorylation 52 2.19 2.10E-5

Regulation of kinase activity 40 3.08 1.89E-7

Regulation of GTPase activity 39 6.43 4.58E-16

Haemostasis 21 4.76 2.18E-6

Platelet activation 17 8.35 4.16E-8
